# Phosphoproteomic Approaches for Identifying Phosphatase and Kinase Substrates

**DOI:** 10.3390/molecules28093675

**Published:** 2023-04-24

**Authors:** Andrew G. DeMarco, Mark C. Hall

**Affiliations:** 1 Department of Biochemistry, Purdue University, West Lafayette, IN 47907, USA; 2 Institute for Cancer Research, Purdue University, West Lafayette, IN 47907, USA

**Keywords:** phosphorylation, post-translation modification, PTM, mass spectrometry, phosphoproteomics, quantitative proteomics, kinase, phosphatase, substrate identification

## Abstract

Protein phosphorylation is a ubiquitous post-translational modification controlled by the opposing activities of protein kinases and phosphatases, which regulate diverse biological processes in all kingdoms of life. One of the key challenges to a complete understanding of phosphoregulatory networks is the unambiguous identification of kinase and phosphatase substrates. Liquid chromatography-coupled mass spectrometry (LC-MS/MS) and associated phosphoproteomic tools enable global surveys of phosphoproteome changes in response to signaling events or perturbation of phosphoregulatory network components. Despite the power of LC-MS/MS, it is still challenging to directly link kinases and phosphatases to specific substrate phosphorylation sites in many experiments. Here, we survey common LC-MS/MS-based phosphoproteomic workflows for identifying protein kinase and phosphatase substrates, noting key advantages and limitations of each. We conclude by discussing the value of inducible degradation technologies coupled with phosphoproteomics as a new approach that overcomes some limitations of current methods for substrate identification of kinases, phosphatases, and other regulatory enzymes.

## 1. Introduction

Protein kinases and phosphatases catalyze the addition and removal of phosphate groups from specific substrate proteins to dynamically regulate diverse biological processes. Phosphorylation is one of the most common eukaryotic regulatory protein post-translational modifications (PTMs), detectable at some level on most proteins. Phosphorylation can affect numerous protein properties, including interactions with other biomolecules, stability, cellular localization, and enzyme activity. The significant fraction of genes encoding kinases and phosphatases (e.g., ~3% of human genes) reinforces the important role of protein phosphorylation in regulating cellular physiology [1]. Fully understanding how individual proteins, protein complexes, or entire biological pathways are controlled by phosphorylation requires knowledge of (1) the amino acid positions modified by phosphorylation, (2) the effect of each phosphorylation event on protein structure/function, (3) the identity of kinases and phosphatases responsible for each modification, and (4) how the activities of those kinases and phosphatases and the resulting levels of phosphorylation sites change under different conditions. One of the most common and powerful tools for studying the phosphoproteome is quantitative liquid chromatography-coupled mass spectrometry (LC-MS/MS). The power of LC-MS/MS for studying the phosphoproteome lies in its ability to accurately identify and quantify the exact sites of thousands of phosphorylation events on proteins in complex biological samples within hours [2,3,4,5,6,7,8]. LC-MS/MS is unrivaled in its speed, sensitivity, and throughput for phosphorylation site identification and quantification.

For most proteomic applications, LC-MS/MS systems consist of a nanoflow HPLC unit coupled in-line to a high-resolution tandem mass spectrometer, with Orbitrap and time-of-flight (TOF) analyzers being the most common due to their superior resolution and fast acquisition rates. Advances in instrument technology, including the introduction of ion mobility spectrometry, development of data-independent acquisition capabilities, and increased sensitivity and scan speeds, have further improved phosphoproteome analyses in recent years [9,10,11,12,13]. This review will not cover LC-MS/MS instrumentation, as this topic has been adequately reviewed in the above references and elsewhere. Phosphoproteomic workflows include a variety of methods for phosphopeptide enrichment [7,14], LC-MS/MS data acquisition and phosphopeptide fragmentation [15,16], phosphopeptide identification and phosphosite localization/scoring [17,18], and quantifying phosphosite abundance [5,6,19,20,21]. While these are important components of phosphoproteomics experiments, they have been reviewed thoroughly in the above references and elsewhere [22,23,24] and are beyond the scope of this review. Instead, we focus our discussion here on the breadth of upstream experimental designs that employ LC-MS/MS and associated phosphoproteomic tools for studying the biological substrates of protein kinases and phosphatases.

LC-MS/MS can be applied in many types of phosphoproteomic experiments to study kinase/phosphatase functions and identify candidate substrates. We group these approaches into three general categories for discussion below. The first category involves methods to perturb the target kinase/phosphatase activity in vivo and measure the consequent changes in the phosphoproteome, thereby revealing specific physiological phosphorylation sites dependent on that target. The second category encompasses methods that rely on affinity capture of (1) enzyme-substrate complexes from biological systems, or (2) in vivo proximity-labeled substrates. The third category covers in vitro enzyme assays using substrate pools derived from cell extracts or synthetic peptide libraries. We try to summarize the strengths and limitations of each category for identifying direct kinase and phosphatase substrates, and emphasize that a combination of multiple approaches is often most effective at achieving this goal. For each category, we point out representative examples from the literature. There are many exceptional phosphoproteomic studies to choose from and we apologize to those groups whose work we were not able to include for the sake of brevity. We conclude by highlighting a general approach in its infancy, the coupling of inducible degradation technologies with proteomics, which may have broad applicability for identifying substrates and characterizing the biological specificity of kinases, phosphatases, and many other regulatory enzymes in dynamic biological processes.

## 2. Methods Involving In Vivo Enzyme Perturbation

**Figure 1:**
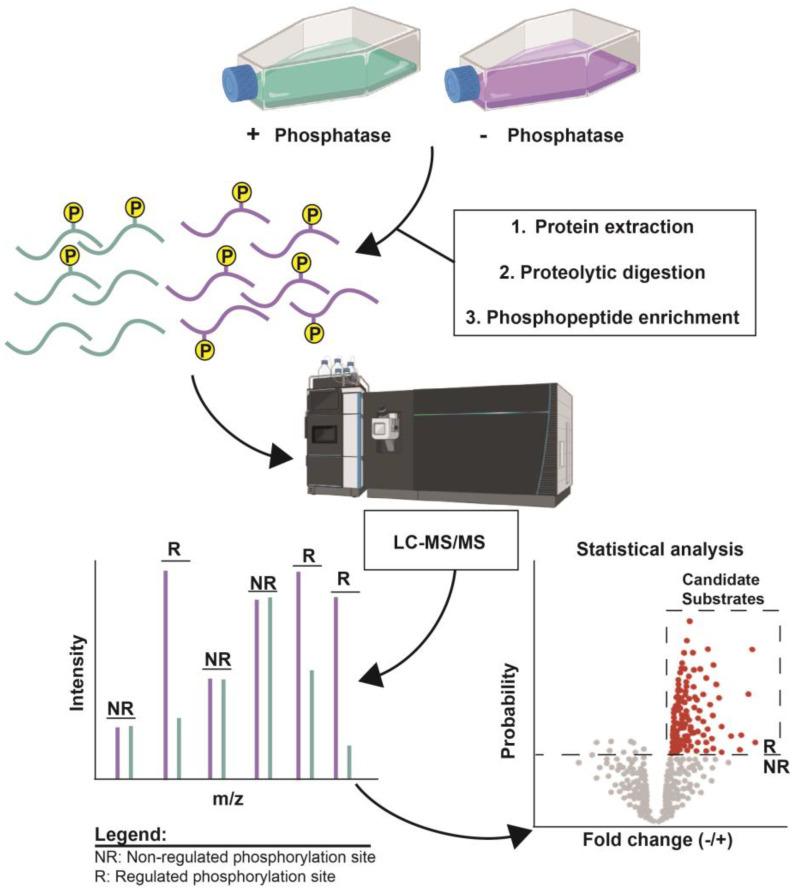
Example experimental design to identify candidate phosphatase substrates by altering target enzyme activity. Biological samples differing only in the activity state of the target of interest (in this case a phosphatase, but it could also be a kinase) are grown and harvested. Metabolic stable isotope labeling of proteins for MS quantification is optional at this step. Proteins are extracted from both samples, digested with a protease like trypsin, and phosphopeptides selectively enriched. Chemical labeling of peptides is another option for stable isotope incorporation for quantification. When stable isotopes are used, the two phosphoproteomes can be analyzed together by LC-MS/MS, with relative differences in peak intensities reflecting sites regulated by the target enzyme. Phosphopeptide sequences are identified using database search software and significant differences in abundance between samples, reflecting candidate substrates, are determined using statistical tools. Created with BioRender.com.

This category includes methods that (1) genetically alter target protein expression, or (2) chemically inhibit target enzyme activity in the natural biological setting. In both cases (Figure 1), the phosphoproteomes associated with normal and altered target activity are quantitatively compared, and statistically evaluated, to identify phosphosites regulated by the target kinase/phosphatase. This provides a list of candidate substrates of the target but does not guarantee that any given regulated phosphosite is a direct substrate. Additional experimentation is required to make such a conclusion. However, chemical inhibition methods are more likely to specifically reveal direct substrates because of the speed with which they act. Stable isotope labeling methods are commonly employed to provide the most accurate quantification of differences between the normal and altered states, and these approaches invariably require some form of phosphopeptide enrichment to maximize coverage of the thousands of phosphorylation sites present in biological systems. These experiments may also include additional variables, such as signaling pathway stimulation or stress treatment, to reveal sites regulated by the target kinase/phosphatase under specific conditions.

- Genetic disruption of target expression.

A common approach to comparing systems with normal and altered target enzyme activity, is to disrupt the endogenous target gene. This completely eliminates target enzyme expression, and the advent of CRISPR gene editing methods has made this approach feasible in a broad range of biological systems. For example, two groups performed large-scale analyses of many kinase and phosphatase gene deletion strains to better understand global protein phosphoregulation in the model yeast *Saccharomyces cerevisiae* [25,26]. Similarly, the Loizou group used CRISPR to disrupt 313 kinase genes in cultured human cells and compared changes in the phosphoproteomes in response to different types of DNA damage in these cell lines [27]. Alternative methods to disrupt expression include regulatable promoters to induce or terminate target gene transcription, and small interfering RNA (siRNA) to selectively degrade target gene transcripts by RNA interference (RNAi) systems. The Kettenbach lab used baculovirus-mediated RNAi to suppress the human protein phosphatase 6 (PP6) catalytic subunit and then performed comparative phosphoproteomics to identify mitotic PP6 substrates, including a subunit of the condensin complex whose phosphoregulation is important for chromosome condensation and segregation [28]. The Westermarck group used siRNA repression to identify protein phosphatase 2A (PP2A) substrates whose recognition is regulated by different PP2A protein inhibitors [29]. An inducible promoter system was used to profile substrates of the essential PstP phosphatase in *Mycobacterium smegmatis*, revealing broad regulation of kinase activity and protein phosphorylation in this species [30]. Galactose-induced overexpression of the fungal-specific Ppz1 phosphatase was used in *S. cerevisiae* to identify candidate substrates based on decreased phosphorylation site abundances in LC-MS/MS analysis [31]. In addition, a tetracycline-repressible promoter was used to identify phosphorylation sites regulated by the calcium-dependent protein kinase CDPK7 in the parasite *Toxoplasma gondii* [32].

- Chemical inhibition of target enzyme activity.

The other general method used to suppress a target kinase or phosphatase activity and measure resulting changes in the phosphoproteome, is chemical inhibition. This can be in the form of specific chemical inhibitors that target individual enzymes or a chemical genetic approach in which different enzymes can be engineered to each respond to a common chemical inhibitor. In the case of kinases, numerous specific inhibitors have been developed, mostly as therapeutic molecules [33,34,35,36]. Some of these inhibitors have been used in proteomic experiments to identify candidate substrates using the same general approach used to compare normal versus altered expression [37,38,39,40,41,42,43,44]. For example, the Gerber lab used a suite of small molecule kinase inhibitors to link candidate substrates to Aurora A, Aurora B, and Polo kinases in mitotic cells [37]. Similarly, Rimel et al. used a specific CDK7 inhibitor coupled with SILAC phosphoproteomics to uncover novel substrates related to transcription and a role for CDK7 in regulation of mRNA splicing [38]. Finally, the Mann lab used MAP kinase and BCR-ABL inhibitors coupled with SILAC phosphoproteomics to evaluate the specific effects of these kinases on the epidermal growth factor signaling pathway [41].

Some kinases have also been successfully targeted using a “bump-and-hole” chemical genetic approach, pioneered by the Shokat laboratory, in which mutation of the gatekeeper residue in the ATP binding pocket accommodates a bulky ATP analog inhibitor that is unable to bind native kinases [45,46,47,48]. Importantly, this “analog-sensitive” mutation often has little impact on kinase activity and function, and a comparison of the phosphoproteome before and after brief treatment with the inhibitor reveals phosphosites specifically dependent on the analog-sensitive kinase. This approach has been particularly effective for identifying substrates of various cyclin-dependent kinases (CDKs), including CDK1 in *S. cerevisiae* [49] and *Schizosaccharomyces pombe* [50], human CDK2 [51], the transcriptional regulator CDK9 [52], and a viral CDK [53]. It has been successful for other kinase families as well [54].

The use of inhibitors in phosphoproteomic studies of phosphatases has been much more limited. There are no broadly applicable chemical genetic approaches akin to the analog-sensitive kinase strategy, and relatively few specific phosphatase inhibitors exist. The potent phosphoprotein phosphatase (PPP) family inhibitors, such as Okadaic acid, Calyculin A, and microcystin-LR, are generally not selective enough for confident identification of substrates of specific PPP holoenzymes. However, the Thibault group was able to combine the selective PP2A activator SH-BC-893 and inhibitors LB-100 and C2-ceramide in a phosphoproteomics study to uncover candidate PP2A substrates and a role for PP2A in regulation of nutrient transporters and actin cytoskeletal remodeling [55]. The protein tyrosine phosphatase (PTP) family has been more amenable to specific inhibitor development [56]. For example, the Shp2 inhibitor, SHP099, was used in phosphoproteomic studies to identify new roles in specific signaling pathways [57,58]. However, there are surprisingly few examples of specific PTP inhibitors used in phosphoproteomic studies to identify substrates, possibly because of the success of PTP substrate-trapping mutations described below for substrate identification.

- Advantages and disadvantages of target perturbation methods.

Although widely used and relatively easy to implement, gene disruptions are limited to non-essential genes. Repressible promoters have the advantage of being applicable to essential genes and allow experimental control over the timing of target suppression. However, target activity is not completely eliminated, and the extent of reduction can be variable. Likewise, RNAi methods exhibit variability in the extent of reduction for different targets. All of these approaches for reducing target expression share a major caveat: the target enzyme is absent or suppressed for relatively long periods of time. In the case of gene deletions, it is permanent, while in the case of repressible promoters and RNAi, the time required for maximum target protein reduction depends on its intrinsic stability. The sustained loss of a kinase or phosphatase using these methods can significantly alter cellular physiology, leading to indirect proteome changes that make it difficult to conclusively identify direct substrates. For example, transcriptional responses to target loss may lead to changes in the expression of other kinases and phosphatases and subsequent changes in the phosphorylation of their substrates. In addition, it may lead to changes in protein abundance through transcriptional or proteolytic mechanisms, complicating the interpretation of phosphorylation site changes. This problem was documented by the Gygi lab, which performed a global phosphoproteomics analysis of 110 kinase and phosphatase gene deletion strains of *S. cerevisiae* and found that 50% of regulated phosphosites were attributable to changes in protein abundance [26]. Approaches have been developed, though, to adjust phosphoproteome abundances based on simultaneously measured changes in the general proteome, independent of the method used to alter target gene expression [25,26,27,28].

Chemical inhibitors have significant advantages in phosphoproteomic studies aimed at kinase and phosphatase substrate identification. First, the use of inhibitors requires no genetic modification to the organism, eliminating the potential for disrupting normal target function. Second, chemical inhibitors typically act very rapidly, minimizing the accumulation of indirect proteome changes discussed in the preceding paragraph. However, there are significant caveats as well. The specificity of many chemical inhibitors is not well-characterized, and one must always be cognizant of possible off-target influences on the results [37,38]. The most significant problem is that highly specific, bioavailable chemical inhibitors simply are not available for most kinases and phosphatases. The analog-sensitive kinase approach provides very high specificity. However, not all kinases are amenable to the creation of functional analog-sensitive variants [59]. Another caveat, unique to many phosphatases, such as the PPP family, is that a single catalytic subunit is often shared by many different holoenzymes that each exhibit unique substrate specificity. Thus, inhibiting the catalytic subunit reveals limited information about the specific substrates of any given phosphatase holoenzyme. In Section 6 below, we highlight an alternative chemical genetic approach, inducible protein degradation, that is more broadly applicable to target protein inhibition and is beginning to see use in phosphoproteomic characterizations of kinases and phosphatases. It circumvents many disadvantages of the target perturbation methods discussed above.

## 3. Methods Involving Affinity Capture of Candidate Substrates

Another broad class of methods involves biochemical isolation and MS identification of proteins physically associated with a kinase or phosphatase of interest from their natural biological system. Many of these methods provide a list of physically interacting proteins but, on their own, do not provide information on regulated phosphorylation sites, and confident identification of direct substrates requires independent validation experiments. Affinity capture methods can be broadly divided into two categories: (1) co-purification of enzyme-substrate complexes; and (2) in vivo proximity labeling approaches.

- Co-purification of enzyme-substrate complexes.

**Figure 2:**
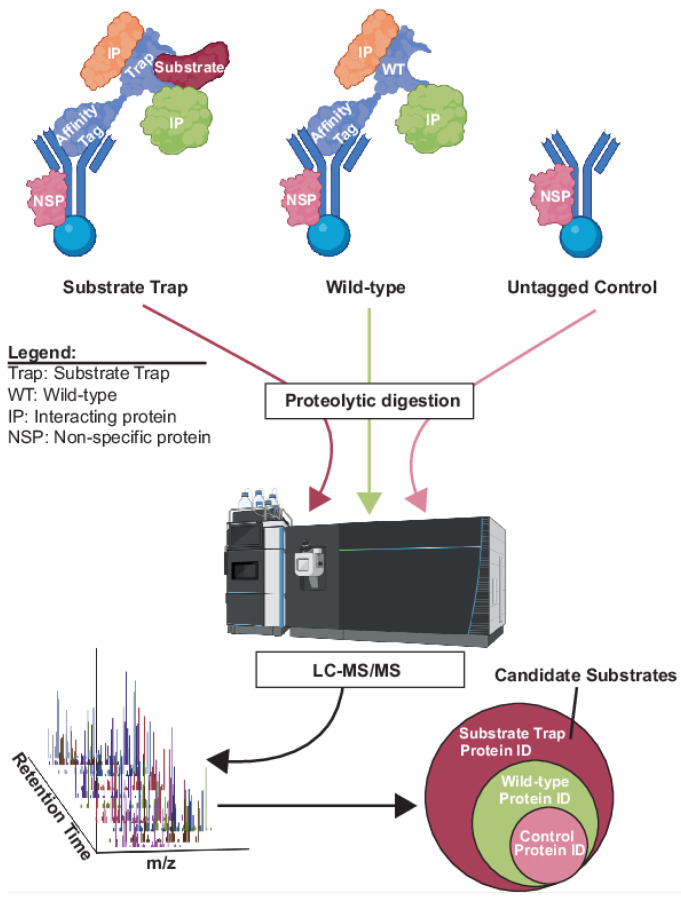
Example experimental workflow for substrate trap AP-MS approach to identify candidate phosphatase substrates. See text for details. The substrate trap and wild-type phosphatases with bound proteins are captured from extracts on antibody or other affinity beads and then digested with a protease prior to LC-MS/MS analysis and protein identification. Other AP-MS methods would have similar workflow, but typically just experimental and control samples. Isotope labeling is not essential, but chemical labeling of peptides can be incorporated if desired. Subtracting proteins identified in the wild-type and control samples reveals candidate substrates. Proteins identified in wild-type but not control reflect non-substrate interaction partners. Created with BioRender.com.

Affinity purification of a protein of interest from a cell extract under physiological conditions, coupled with mass spectrometry identification of co-purifying proteins (AP-MS), is a general approach to identifying interaction partners and can be applied to some kinases and phosphatases to generate candidate substrate lists [60,61]. In AP-MS experiments, a control sample lacking the AP target but processed identically to the experimental sample is required to distinguish specific interactors from the non-specific background [62,63]. These methods are not strictly dependent on accurate quantification afforded by stable isotope labeling and are commonly performed using label-free quantification. Conventional AP-MS has limited utility for substrate identification of most kinases and phosphatases because the enzyme-substrate interactions are often not stable enough to persist throughout the lengthy biochemical isolation process. The use of “substrate trap” mutants, originally described by the Tonks lab for PTP1B [64], has been a valuable solution to this problem for the PTP family, making AP-MS methods a viable option to identify candidate substrates (Figure 2). The “substrate trap” strategy involves mutation of one or more active site residues in PTPs that blocks catalysis without disrupting substrate binding [64,65]. The resulting stabilization of the enzyme-substrate complexes allows their biochemical isolation and analysis by MS. Because AP-MS does not specifically reveal substrate interactions, researchers commonly compare interaction partners of wild-type and substrate trap PTP enzymes, with the idea that substrate abundance will be higher in the substrate trap AP, but other interaction partners will be equal (Figure 2). This substrate trap strategy has been effectively applied to numerous PTP family members, including dual-specificity phosphatases like Cdc14 [66,67,68,69,70,71,72].

Unfortunately, analogous substrate trap mutations do not work with other phosphatase families or with kinases. However, a substrate trapping method was developed by the Bollen lab for the PPP family member PP1, in which a reduced activity catalytic subunit mutant is genetically fused to one of its many substrate recruiting subunits [73]. This allows biochemical isolation and MS characterization of enzyme-substrate complexes for a specific PP1 holoenzyme. Another method to stabilize enzyme-substrate complexes during the AP process is the application of chemical crosslinkers. This approach has been used for kinases by the Phlum lab, which developed an ATP analog containing a photoactivable group that allows covalent linkage of enzyme and substrate during the phosphorylation reaction [74,75,76]. AP of the target kinase then allows MS identification of covalently linked substrates. However, since ATP is not cell permeable, this approach is limited to cell lysate applications.

- Proximity labeling.

**Figure 3:**
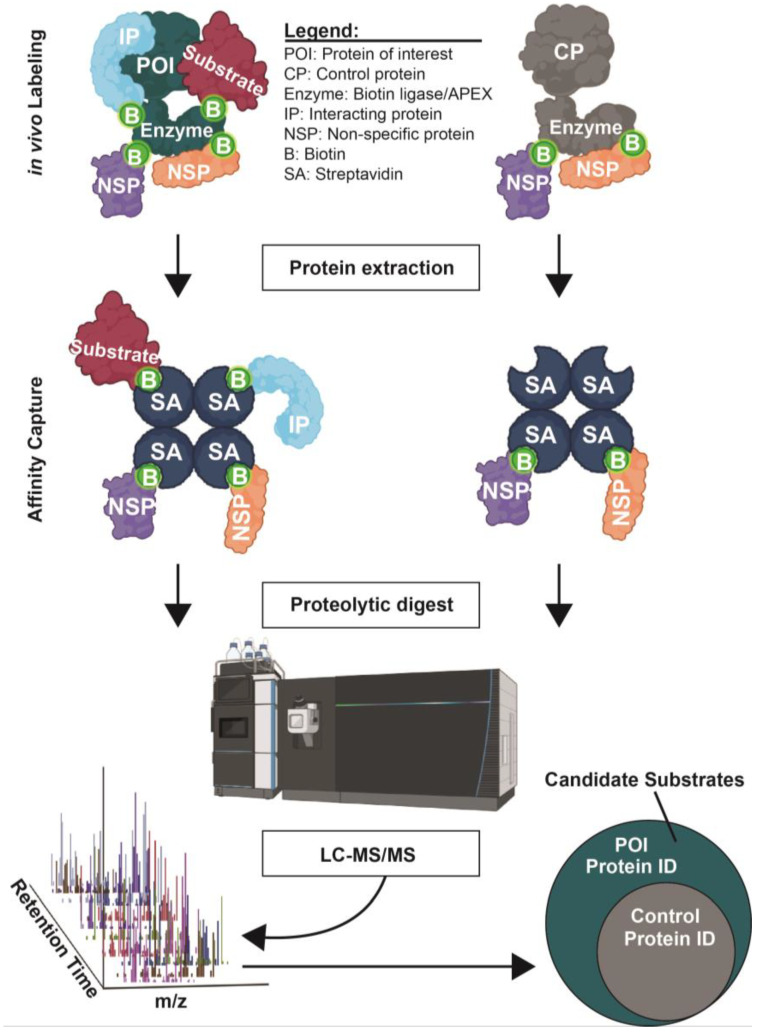
Example experimental workflow for in vivo proximity labeling to identify candidate kinase/phosphatase substrates. See text for details. The target protein of interest (POI) is expressed in the biological system as a fusion with the engineered catalytic domain of a biotin ligase or peroxidase (labeled “Enzyme”), resulting in biotinylation of proteins in the immediate proximity. An appropriate negative control protein fused to the same enzyme is essential for measuring and eliminating non-specific background biotinylation. Streptavidin affinity capture is used to selectively isolate biotinylated proteins from both samples for LC-MS/MS and protein identification. Created with BioRender.com.

Proximity labeling approaches are based on the in vivo covalent labeling of proteins with biotin in the immediate vicinity of a target protein of interest. They require fusing the target gene with the coding sequence for an enzyme, either a peroxidase or biotin ligase, which will generate a reactive biotin moiety from a provided substrate to covalently react with specific functional groups within a very short radius of its active site [77]. For example, the APEX2 [78,79] approach uses a target protein-ascorbate peroxidase 2 fusion and the substrates biotin phenol and H_2_O_2_ to generate a biotin phenoxyl radical that labels nearby proteins, preferentially on Tyr, Trp, Cys, and His sidechains. Similarly, TurboID uses an engineered biotin ligase domain fused to the target to generate biotin-5′-AMP from ATP and free biotin, which then efficiently reacts with primary amines on nearby lysine sidechains [80,81,82]. The effective radius of labeling is roughly 10 nm due to chemical quenching effects and, therefore limited to closely associated proteins, e.g., substrates or other interaction partners. The biotinylated proteins are purified using streptavidin beads and identified by LC-MS/MS (Figure 3). This approach has been combined with the substrate trap method to help identify PTP1B substrates [70]. APEX2 has been used to identify candidate substrates of human MAP kinases [83,84], and TurboID has been used to identify candidate substrates of kinases like *Drosophila* Alk [85] and *Arabidopsis* BIN2 [86], among others. As in AP-MS, a negative control in which the target is replaced by a control protein (e.g., a mutant target unable to bind substrates) fused to the labeling enzyme is needed, to account for non-specific background labeling (Figure 3).

- Advantages and disadvantages of affinity capture methods.

A general advantage of affinity capture methods is that they reflect physical associations with the target kinase or phosphatase, as would be expected for substrates of an enzyme. On the other hand, most of these approaches do not distinguish substrates from other types of protein-protein interactions without additional experimentation, and most do not exclusively report direct interactions. Even with the use of cross-linking or substrate-trapping approaches, the transient nature of enzyme-substrate interactions can limit their detection. Moreover, all AP-based approaches, which require biochemical recovery of complexes from soluble cell extracts, have the caveat that identified binding partners may not accurately reflect real in vivo interactions. This problem is overcome with proximity labeling methods that capture transient interactions like many enzyme-substrate contacts as they occur naturally in the biological system. Proximity labeling does not require the enzyme-substrate complex to survive biochemical purification. Downsides to proximity labeling include high background in some systems and the lack of bias for labeling substrates over other interaction partners or nearby proteins, which necessitates additional experimentation to convincingly identify new substrates. Finally, a common disadvantage of affinity capture approaches to substrate identification is that they provide little information on phosphorylation sites. They are good at revealing the identity of candidate protein substrates, but not the exact sites of modification.

## 4. Methods Involving In Vitro Enzyme Reactions

As an alternative to characterizing kinase/phosphatase substrates using the in vivo proteomic experiments summarized above, these enzymes can be analyzed in vitro with substrate pools generated from cell extracts or synthetic peptide libraries to facilitate substrate identification. In this case, LC-MS/MS is used to detect and quantify reaction products generated by the enzyme of interest over time. In vitro experiments can be beneficial for generating relative reaction rates against different substrates, providing detailed information about the intrinsic specificity of an enzyme that is more difficult to glean from in vivo proteomic experiments where direct and indirect substrates are often hard to distinguish. Intrinsic specificity information can then be useful for predicting substrates and prioritizing candidate substrate lists generated by in vivo phosphoproteomic experiments.

- In vitro experiments using cell extracts.

When in vitro experiments use substrates generated from cell extracts, reactions can either be conducted at the intact protein level, or the extract proteins can first be converted to a peptide pool, e.g., by digesting with a protease, prior to adding a purified kinase/phosphatase of interest. While intact protein substrates have obvious advantages of retaining native structures that may be important for kinase/phosphatase recognition, the activity of endogenous kinases and phosphatases presents a significant challenge. Several creative solutions have been developed to address this. One approach for kinases involves inhibition of endogenous activities in the extract with the covalent inhibitor 5′-[p-(fluorosulfonyl)benzoyl]adenosine (FSBA) [87,88], which is then removed by desalting before adding the purified kinase of interest. This approach ensures any measured effects are directly attributable to the added kinase. Confidence in the assignment of identified sites to the kinase of interest was further enhanced in this strategy by using heavy isotope-labeled ATP-γ-^18^O_2_ as a cofactor in the kinase reaction to distinguish pre-existing sites from those added in vitro by mass shift [89]. A different approach to distinguishing specific substrates of the added kinase in FSBA-treated extracts is the use of an ATP-biotin cofactor, which can result in biotinylation of substrates of some kinases, allowing for their capture on streptavidin beads and subsequent LC-MS/MS identification [90]. Another strategy, developed in *S. cerevisiae*, employed cryogenic lysis and rapid affinity isolation of a kinase under physiological conditions immediately followed by on-bead kinase reaction with ATP-γ-^18^O_4_ [91]. The approach was applied to CDKs and revealed that kinase-substrate complexes were at least partially maintained during the rapid, gentle biochemical isolation, and that “heavy” phosphopeptides identified by subsequent LC-MS/MS analysis accurately reflected direct substrates of the target kinase. A related method involves passing extracts through a recombinant kinase affinity column to enrich for interacting substrates, followed by on-bead in vitro kinase reaction and LC-MS/MS identification of in vitro phosphorylation sites [92]. A third approach for kinases employs the analog-sensitive ATP binding site gatekeeper mutation described above for in vivo kinase inhibition proteomics [59,93]. In short, purified analog-sensitive kinases are added to protein extracts in the presence of ATP-γ-S, resulting in specific thiophosphorylation of the target kinase’s substrates. The thiophosphorylated proteins can then be either directly captured after alkylation by immunoaffinity using anti-thiophosphate ester antibodies and identified by LC-MS/MS [94], or digested with a protease and thiophosphorylated peptides covalently captured on iodoacetyl resin, released by oxone-mediated hydrolysis and analyzed by LC-MS/MS [95,96,97]. The latter provides phosphorylation site information and therefore is preferential.

Unfortunately, covalent pan-phosphatase inhibitors, similar to FSBA for kinases, do not exist. Nonetheless, several groups have developed extract-based in vitro proteomic methods for the characterization of specific protein phosphatases. Substrates of the abundant heterotrimeric PP2A phosphatase family have been profiled in cell extracts using protein inhibitors. For example, the Nilsson lab identified specific substrates of PP2A-B56 and PP2A-B55 in human mitotic cell extracts after brief incubation with either a synthetic peptide containing the optimal B56 substrate docking motif LxxIxE to competitively inhibit substrate binding, or a thiophosphorylated version of the known PP2A-B55 inhibitor Arpp19 [98,99]. The brief inhibition was sufficient to alter phosphorylation levels on substrates, presumably due to persistent opposing kinase activities in the extracts. To compare the intrinsic specificity of PP1 and PP2A catalytic subunits, the Köhn lab used mitotic extracts generated after treatment with a low concentration of the broad-spectrum PPP family inhibitor, Calyculin A, and then added relatively high concentrations of the purified catalytic subunits for a defined time prior to quenching and comparative LC-MS/MS analysis [100].

- In vitro experiments using peptide substrate pools.

An alternative approach to circumvent the problem of endogenous activities in protein extracts is to first reduce proteins to smaller polypeptides via protease treatment. This approach maintains the natural collection of phosphorylation site sequences recognized by kinases and phosphatases, but effectively eliminates the background of endogenous kinase and phosphatase activities that complicate phosphoproteomic experiments. An example application was the use of Lys-C-generated mitotic phosphopeptides enriched from human cell extracts to identify sites efficiently dephosphorylated by affinity-purified PP2A-B56 [98]. This nicely augmented in vivo results obtained by overexpressing a PP2A-B56 inhibitor. The Tao lab developed an in vitro approach to profile kinase substrates using peptide pools generated from dephosphorylated and protease-treated protein extracts that are subsequently treated with purified kinases and analyzed by LC-MS/MS [88,101].

Synthetic peptide libraries have been useful in characterizing the specificity of purified protein kinases, for example, by detecting ^32^P incorporation into peptide spots on membrane arrays [102,103,104] because amino acids surrounding the phosphorylation site are the primary specificity determinants for many kinases. In principle, MS provides an ideal readout for large collections of peptide substrates reacted with a kinase or phosphatase of interest, but this approach has seen more limited use. Using synthetic peptide substrates for kinases or phosphatases provides the ultimate in experimental control, for example, allowing accurate calculation of rate values like *k_cat_*/*K_M_* and direct comparison of different substrate sequences. This helps define intrinsic specificity and the substrate features that contribute to it [105,106,107], and can be used to define consensus recognition motifs for the catalytic site of an enzyme that, in some cases, can allow bioinformatic prediction of new substrates and functions [108]. For kinases, one of the first implementations of synthetic peptide libraries was termed the Kinase-Client assay, or KiC, and involved a collection of synthetic peptide sequences based on known in vivo phosphorylation sites that were reacted with purified recombinant kinases to match them with substrate sites [109]. The Köhn lab developed a method for large synthetic phosphopeptide library generation that randomized specific positions around the phosphorylation site and was used to compare the intrinsic specificities of the catalytic subunits of PP1 and PP2A [100,110]. Synthetic phosphopeptide libraries should be useful for the specificity characterization of PTP family members because, like kinases, key specificity determinants are often adjacent to the target phosphorylation site [111,112].

- Advantages and disadvantages of in vitro phosphoproteomic approaches.

Perhaps the most significant advantage of in vitro experiments for kinase/phosphatase substrate identification, is the ability to eliminate or minimize the activity of other enzymes to ensure observed effects are directly attributable to the enzyme of interest. Closely related to this, in vitro experiments are better suited for rate measurements that allow different substrates to be compared and the intrinsic specificity of an enzyme defined. That specificity can aid the interpretation of in vivo experimental results to help identify true biological substrates. However, there are numerous disadvantages of in vitro experiments as well. Generally speaking, in all in vitro experiments, at least some important biological context is lost or perturbed, e.g., cellular localization, interacting molecules, or protein concentrations, which can alter substrate recognition. In particular, the concentration of the kinase or phosphatase used for in vitro experiments is often much higher than the physiological concentration, which can result in artificial activity towards some substrates. For peptide experiments, the 3-dimensional structure of substrates is missing, which may be crucial for substrate recognition by the enzyme of interest. Because many kinases and phosphatases rely on docking interactions distal from the substrate phosphorylation site, peptides will often not contain the full complement of substrate recognition features. Overall, it is particularly important that candidate substrates identified solely by in vitro phosphoproteomics be validated with orthogonal experiments.

## 5. Combining Multiple Methods Can Improve Substrate Identification Confidence

From the summaries above, it is evident that all individual phosphoproteomic methods aimed at identifying kinase and phosphatase substrates have distinct limitations. Consequently, the most rigorous characterizations and confident identifications of bona fide substrates apply multiple methods with complementary strengths. Specifically, combinations of in vivo and in vitro methods can allow researchers to overcome ambiguities arising from the application of a single method and develop more refined, high-confidence candidate substrate lists. We highlight just a few examples of studies that have effectively implemented a combination of proteomic methods to identify novel kinase or phosphatase substrates. In addition to providing increased confidence in substrate assignments, these studies emphasize that using the overlap of independent experimental approaches significantly decreases the size of the candidate list for subsequent biological validation.

Kruse et al. [98] used inducible expression of an engineered PP2A-B56-specific inhibitor protein in HeLa cells to identify in vivo phosphorylation sites dependent on PP2A-B56 activity. To better understand which sites are direct substrates of PP2A-B56, the authors applied two in vitro phosphoproteomic experiments. First, they reacted a pool of phosphopeptides generated by protease digestion of a cell extract with purified PP2A-B56 and monitored the kinetics of phosphopeptide dephosphorylation by LC-MS/MS. Second, they added a synthetic B56-specific peptide inhibitor to cell extracts and monitored the effect on dephosphorylation of sites on intact proteins by LC-MS/MS. Collectively, these approaches provided novel insight into substrate recognition mechanisms of PP2A-B56 and the identification of novel biological substrates.

Kinase assay linked with phosphoproteomics (KALIP) uses cross-validation between in vivo and in vitro phosphoproteomic experiments to provide more confident direct substrate identifications [88,101]. KALIP employs (1) an in vitro kinase reaction with a phosphatase-treated peptide library derived from cell lysates, and (2) an in vivo phosphoproteome comparison of cells with and without overexpression of the target kinase. The in vitro experiment reveals substrate sites capable of direct phosphorylation by the kinase, whereas the in vivo experiment reveals sites that can be influenced by the kinase in the natural setting. Sites identified in both approaches have a higher probability of being direct substrates. This approach should be applicable to phosphatases as well, and indeed, this is essentially what the Nilsson group did in characterizing PP2A-B56 [98].

Zhu et al. [113] combined three approaches to confidently identify Shp2 phosphatase substrates: (1) comparison of phosphopeptide abundances identified by AP-MS of wild-type and substrate trap mutant Shp2 expressed in cultured human cells, (2) comparison of the pTyr phosphoproteomes of cells expressing either wild-type or inactive Shp2 after EGF stimulation, and (3) in vitro Shp2-catalyzed dephosphorylation of phosphopeptides generated from proteins co-purifying with the substrate trap Shp2.

Malik et al. identified novel substrates of the serum- and glucocorticoid-regulated kinase 3 (SGK3) by relying on the overlap of two orthogonal approaches to perturbing SGK3 activity in vivo [114]. They conducted quantitative phosphoproteomic comparisons of (1) wild-type and SGK3 knockout HEK293 cells, and (2) untreated and SGK3 inhibitor-treated cells, each following stimulation with IGF1. The overlap of these two experiments and a filtering step for the known phosphorylation consensus sequence of SGK3, reduced the candidate substrate list to sites on just five proteins, with four phosphosites subsequently validated by in vitro kinase assays.

## 6. Coupling Inducible Degradation with Phosphoproteomics to Define Kinase and Phosphatase Substrates

In summary, there are a wide variety of proteomic methods applied to the difficult problem of identifying the physiological substrates of kinases and phosphatases. A persistent challenge is distinguishing direct substrates from other phosphorylation sites that are indirectly affected by the perturbation of the target enzyme in vivo. This problem is most acute when using methods resulting in long-term loss of target enzyme function, such as gene disruptions and RNAi-mediated transcript knockdowns. For that reason, the use of specific chemical inhibitors coupled with phosphoproteomics has a clear advantage in its speed of action, allowing the researcher to survey phosphoproteome effects on a short time scale before the accumulation of downstream, indirect changes. The lack of well-characterized, highly specific inhibitors for most kinases and phosphatases prevents the widespread use of this approach. We point out that inducible protein degradation technologies can fill this gap, providing a suitable (and in some ways superior) surrogate to chemical inhibitors for the rapid inactivation of kinase and phosphatase (and other regulatory enzyme) targets, allowing characterization of the immediate phosphoproteome changes using quantitative LC-MS/MS.

**Figure 4:**
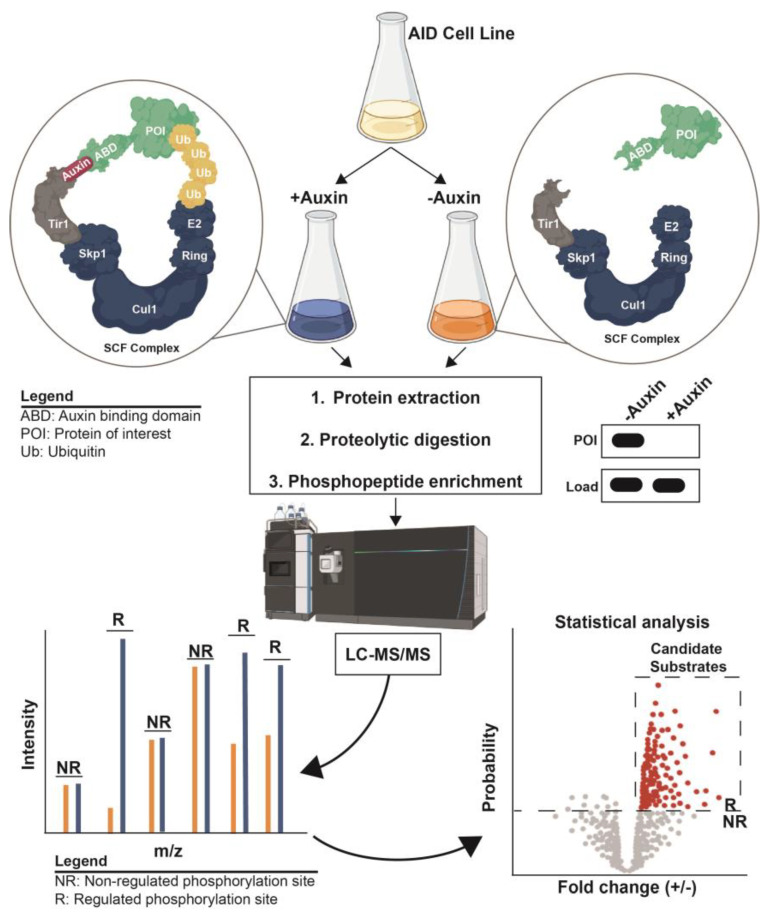
Experimental design of AID-based phosphoproteomic experiment to identify candidate kinase or phosphatase substrates. The biological system is engineered to express a plant Tir1 F-box protein, and the target of interest fused to a plant ABD (the “AID Cell Line”). Addition of auxin triggers rapid polyubiquitylation and proteasomal degradation of the target, mediated by interaction of Tir1 with the endogenous SCF ubiquitin ligase (see cartoon immunoblot at right). Two samples (+/− auxin) are collected, proteins extracted and digested, and phosphopeptides enriched, followed by LC-MS/MS analysis to identify regulated phosphosites. Either metabolic or chemical isotope labeling can be used for direct sample comparison in a single LC-MS/MS run. Created with BioRender.com.

Multiple inducible degradation technologies have been developed, but they all share the fusion of a degron sequence to the target gene of interest that promotes the rapid proteasomal degradation of the fusion protein in response to the addition of a chemical reagent or a change in experimental condition (e.g., temperature or light). These systems are thoroughly reviewed elsewhere [115,116,117,118]. Here, we focus on just the auxin-inducible degradation (AID) system [119], which appears to be the most convenient, commonly used, and widely applicable inducible degradation technology, and a system that research groups are beginning to combine with phosphoproteomics. The AID system, based on the natural function of the plant hormone auxin, requires fusion of the target gene to the coding sequence of a plant auxin-binding domain (ABD) and the expression of a plant Tir1 F-box protein (Figure 4). Auxin acts as a molecular glue that mediates interaction of ABD and Tir1, resulting in the recruitment of ABD to the SCF E3 ubiquitin ligase for polyubiquitylation and proteasomal degradation. In many model organisms, such as *S. cerevisiae*, near-complete target degradation can be achieved within 10–15 min of auxin addition to liquid cultures [119]. Thus, auxin essentially acts as a universal, highly specific chemical inhibitor for any target of interest. While natural auxin can have off-target physiological effects in some species [120,121,122,123], a newer version of AID called AID2 used the bump-and-hole approach to create mutant Tir1 that responds to extremely low concentrations of a bulky synthetic auxin analog [123,124], greatly reducing the chance of off-target effects. AID and AID2 appear to be applicable in all major eukaryotic clades with the exception of plants, having been established in budding and fission yeasts, *Plasmodium falciparum*, *Drosophila melanogaster*, *Caenorhabditis elegans*, mice, cultured human cells, and others [123,124,125,126,127,128,129].

The Reiter group recently published the first successful implementation of AID coupled with phosphoproteomics to reveal novel insight into the substrates and functions of a kinase or phosphatase, targeting the PP2A-Cdc55 phosphatase in the osmotic stress response of *S. cerevisiae* [130]. Importantly, this group demonstrated that rapid Cdc55 degradation mediated by AID effectively reproduced the phosphoproteome changes observed with *cdc55* gene deletion while avoiding transcriptional “adaptation” to prolonged loss of Cdc55 function in the deletion strain and helping clarify the critical early role of PP2A-Cdc55 inhibition by endosulfine proteins in the signaling response to osmotic stress. PP2A-Cdc55 was also targeted using AID to reveal potential substrates in *S. cerevisiae* by another group [131]. AID phosphoproteomics has also been applied successfully to the human Polo kinase Plk1, comparing the effects of chemical inhibition to AID-mediated inhibition, with a high correlation of down-regulated phosphosites observed between the two approaches [132]. Furthermore, AID-coupled phosphoproteomics has been applied to human Protein Phosphatase 6, demonstrating the ability to identify specific changes in the phosphoproteome and novel PP6 substrates without significant changes to the general proteome [133].

Inducible degradation offers several key benefits for proteomic identification of direct substrates of kinases, phosphatases, and other regulatory enzymes. First, the rapid depletion achieved with AID should greatly reduce the background of phosphoproteome changes resulting indirectly from long-term adaptation and transcriptional responses to target gene deletion, transcriptional repression, or siRNA-mediated transcript knockdown. Related to this, as long as the ABD tag does not compromise target protein function, AID ensures normal physiology and target gene regulation up to the point of auxin addition. Transcriptional repression systems, for example, require the use of artificial promoters that eliminate natural control of target expression. AID is amenable to almost any experimental condition, requiring only the need to treat the biological system with auxin, and has been validated in whole animal models [123,126,127], making it broadly applicable. Finally, AID provides exquisitely specific inhibition of the desired target and has minimal non-specific physiological effects, particularly the newer AID2 system [123].

## 7. Conclusions and Future Perspective

Diverse methods have been developed to identify phosphoproteome changes dependent on specific kinases and phosphatases, and to identify candidate substrates interacting with kinases and phosphatases. However, the ability to unambiguously identify direct substrates of kinases and phosphatases remains a challenge and almost always requires additional orthogonal experimental validation. Workflows that minimize the list of candidates for validation are highly valuable. Reducing the list of candidate substrates that require validation can be achieved in a number of ways, including combining in vivo phosphoproteomic approaches with in vitro characterization of kinase/phosphatase specificity. Phosphoproteomic methods that achieve rapid in vivo target inactivation, such as specific chemical inhibition and inducible degradation technologies, have significant advantages in substrate identification by minimizing indirect proteome and phosphoproteome changes. Inducible degradation technologies like AID are relatively easy to implement and can be applied to almost any target protein of interest in many different experimental systems to achieve rapid suppression of target function. Moving forward, coupling inducible degradation systems with phosphoproteomics should therefore be a common and effective strategy for the confident identification of direct substrates of kinases, phosphatases, and other regulatory enzymes.

## Data Availability

Not applicable.
